# Elements of Pathology

**Published:** 1848

**Authors:** Alfred Stille

**Affiliations:** Lecturer on Pathology and the Practice of Medicine in the Philadelphia Medical Association, &c. Being No. 2 of the Medical Practitioner’s and Student’s Library


ARTICLE I.

*Elements of Pathology.* By ALFRED STILLE, Lecturer on Pathology and the Practice of Medicine in the Philadelphia Medical Association, &c. Being No. II of the Medical Practitioners and Students' Library. pp. 483. Phila.: Lindsay & Blakiston. 1848. (From the Publishers.)

THE subject of this volume is one that has, within the last few years, deservedly attracted an increased share of the attention of the profession. It is impossible that a physician should understand special diseases, without being familiar with the general principles of pathology; and as there is no concise work on the especial subject, in our language, save, perhaps, a translation of Chomel's work, that has recently been published in Boston, which we have not yet seen, it cannot be said that the work is uncalled for. But, of late, this question—whether a necessity exists or not for a new work—seems entirely to have been laid aside; and we may be accused of being behind the times for mentioning it, as book after book is made, of late, from precisely the same materials; shown up, to be sure, in a new style in each successive issue, without the question being raised of its necessity; there, certainly, being room for all new works that will sell. And, after all, we are not sure but this is correct; for, after we have gathered facts, for a long time, from an author, his style becomes familiar and dry, while, if they are given by another, they may have new interest. We, on this account, and for the reason that no one man can become pre-eminently qualified to teach in more than one or two departments of the profession, are decidedly opposed to the system which formerly obtained, but is going into disrepute, of having a whole medical library from the same author.

The system adopted by our publishers, of selecting an author for each subject, will give greater research and variety in style, thus furnishing grounds to look for a good and an interesting library.

The authors are all American, too—no small reason why the series should be patronized, as it is time we were becoming more national in this respect.

But to return to the work before us. We really believe it to be opportune in its appearance, and a most valuable treatise. Dr. Stille is a fluent and chaste writer, and is extensively familiar with the subject upon which he has written.

To show some parts of the work to our readers, and thus, by giving them a taste, sharpen their appetites for a full repast, when they shall purchase it, we proceed to make extracts and notice the manner in which each branch of the subject is treated.

The work is introduced by an excellent essay on the subject of medical truth, setting forth clearly, its nature, the difficulties in the way of arriving at it, and the only mode of useful and profitable investigation; this being by patient, correct, systematic, and extensive observation.

It is divided into four parts—the first treats of "Ætiology;" the second, of the "General Phenomena, Theory, and Classification of Diseases; General Diagnosis and Prognosis;" the third of "Semiology;" and the fourth, of "General Morbid Anatomy."

In the first part, are considered, as general predisposing causes, the influences of temperature, heat, cold, the seasons, the climates, cold, temperate, and warm, and acclimation. As special predisposing causes—hereditary predisposition, age, sex, temperament, idiosyncracy, habits of life and profession, food and drink, dress, fatigue, previous disease, the position of organs, &c., and sympathy. As general and special exciting causes—cold, pain and mental emotion, mechanical and chemical causes, and venom. As the specific causes of disease—infection, contagion, medical constitution, and endemic and epidemic influences.

In reference to heat, the author says, correctly, its deleterious influence is principally exerted when accompanied with humidity:

"In the latter case, the disposition to inaction which heat alone creates, is sensibly augmented; the skin, no longer able to part with the moisture of the body, and by its evaporation, to maintain a tolerable temperature, is continually bathed in perspiration; the fluid which would otherwise have escaped from this emunctory, is poured forth by the mucous membrane of the bowels, and the various forms of intestinal flux, diarrhœa, and dysentery, prevail. It is this association of atmospheric influences to which must be chiefly attributed that most fatal scourge of infancy in this country, the summer complaint, or *cholera infantum*. It is the same, also, which appears to be a principal element in the generation of those pestilential maladies which devastate the neighborhood of marshes, and certain other moist localities, although, as will be more fully shown hereafter, other agencies contribute to, and indeed determine this result. It is remarkable, that although hot, dry air predisposes to cerebral, cutaneous, and intestinal inflammations, the lungs should be nearly always exempt from disease in such an atmosphere, and scarcely less so in warm, and moist weather."

The necessity for activity in the functions of respiration for the production of heat, being absent, by which the lungs enjoy immunity from excitement, may account for this.

In reference to cold, our author says:

"In general, dry, cold air imparts to diseases an acute inflammatory type, while, on the other hand, a cold, moist atmosphere predisposes to chronic affections, or to those acute disorders which are peculiarly liable to become chronic, such as rheumatism and bronchitis; dropsies, with all their various organic causes, and all the forms of tubercular disease, are ordinary effects of this cause. In a word, the combination of cold with moisture is more prolific of disease than any other cause, or union of causes whatever."

Passing over the very judicious remarks of our author under the several heads above enumerated, we direct attention, more particularly, to his observations on the subject of infection.

Under this head, the author takes decided ground in favor of the doctrine of marsh malaria as the cause of remittent and intermittent fever, and other diseases, as is seen by the following extract:

"The insalubrity of the neighborhood of marshes, particularly on the side exposed to the wind sweeping over their surface, has been known in all ages, and its reality proved by the restoration of the sickly district to health, on the water of the marsh being carried off by drainage. In fact, wherever a rich, wet, and low soil, abounding with vegetable and animal matters undergoing decay, is exposed to a powerful sun, after repeated irrigations of inundations, remittent and intermittant fevers will prevail, and of a more malignant type as the heat is more intense. It is probably owing as well to the vast number of the exuviæ of insects which crowd the salt-water marshes, as to the high temperature of the air, of intertropical countries, that the mortality of the diseases occasioned by them is owing. But even in these situations, it is not so much during the heat of day, as after nightfall, that the infection is active, a circumstance which has been plausibly explained by supposing that the deleterious emanations being mixed with the moisture of the air, are kept diffused through it during the day, but when the coolness of evening succeeds, descend to the lower strata of the atmosphere, where they of course act with redoubled vigor.

"When the infectious locality is of small extent, and so situated that the effluvia accumulate within it, their power of generating disease is rendered so intense as sometimes to prove fatal almost immediately. This is especially true of emanations from putresent matter, such as is collected in sewers, privies, &c., but almost as rapid an action has been observed in the case of paludial exhalations. Pringle, in his work on diseases of the British army in Holland, relates that among the foraging parties sent out early in the morning, when the meadows and marshes on each side of the road were covered with a thick fog of offensive smell, several men were so suddenly taken with a phrensy, as to throw themselves from off their trusses into the water, imagining they were to swim to their quarters. But in general, centers of malarious infection are not of so malignant a nature. Their influence diminishes rapidly with the distance from them, even upon the surface of the earth, and still more rapidly above this level; on the other hand it is augmented wherever anything tends to arrest and confine the vitiated air, such as cellars, the courts and lanes of towns, and narrow valleys.

"A high temperature tends to produce malarious diseases, chiefly when the air is loaded with moisture, a fact which goes far to favour the opinion that the infectious cause is something substantial, which cannot be supported and carried about in so rare a medium as dry air. It is further corroborated by another fact, viz.: that a calm state of the atmosphere is most favorable to the prevalence of malarious disorders, while a strong wind, especially if it be cold, abates or even puts an end to them. It may be added to these illustrations, that a wind blowing over an infected district may cause the prevalent disease to be experienced in regions where it was before unknown; that if a hill be situated in the direction towards which the wind sets steadily for some time, the people living upon the side nearest the infected locality are more likely to suffer than those who inhabit the intermediate country, and the residents on the further side of the hill may escape the noxious influence altogether. In the same manner walls, houses, running streams, long and winding streets, &c., have been known to form barriers to the progress of infectious disease in one or more directions."

The following quotation is what the author says on the specific character and modus operandi of miasm:

"By condensing the vapours arising in certain malarious districts, small portions of mucus, containing animal matter, ammonia, and hydrochlorate and carbonate of soda, have been obtained. This discovery would appear to countenance the hypothesis of Varro, that marshy emanations contain innumerable and invisible insects. Dr. Holland has, in our own time, revived this hypothesis, which he has illustrated in a style no less elegant than ingenious. Some maintain that heat and moisture, followed by a low temperature, conditions which do certainly exist in the neighborhood of marshes, are sufficient to account for the phenomena of malarious diseases; and however utenable such a position may be, it must be admitted that these conditions of temperature and humidity may act as concurrent causes, by diminishing the power of the system to resist the influence of the miasm which is alone capable of exciting the diseases in question.

"There has been a great deal of discussion, and there is still a great deal of doubt, in regard to the manner in which infectious principles invade the economy. It is evident that there are three possible modes by which they may obtain entrance; through the skin, the alimentary canal, and the lungs. The cutaneous surface may be pretty safely considered as offering a barrier to their passage, so long as the epidermis is intact. The experiments of Bichat, which appeared to show that the odorous particles of a dissecting-room could be taken into the system through the skin, so as to be perceptible in the gas discharged from the bowels, are open to several objections; and those of Collard de Martigny, which seemed to demonstrate that the poisonous effects of carbonic acid gas might be produced by its absorption through the skin, have been contradicted by the results of others, conducted in the same manner. The mucous membrane of the bowels is protected, by its position, from contact with the atmosphere and the morbific particles suspended in it, except so far as these latter may become entangled in the food and drink; but there is neither any direct evidence of the reality of this mode of action, nor, judging from the small quantity of material that could be conveyed in the manner supposed, any probability in its favor.

"There remain, then, the lungs; and nearly all modern authorities agree that it is chiefly through them that infectious agents are introduced into economy. It is well remarked by Dr. Copland, that upon the respiratory surfaces the air may be said to undergo a process of digestion, certain elements or portions of it entering into the circulation, and certain others being given off which have served their purpose in the economy. This writer, however, considers that the morbid agents received into the lungs make their impression upon the organic system of nerves, and thus lay the foundation of the subsequent disease. Without denying the reality of this influence, in view of the very rapid effects sometimes produced by malaria, and of which an illustration has been given on a preceding page, it would appear more worthy of credit that the pernicious substance is absorbed into the blood, which it more or less quickly vitiates, and thus occasions the subsequent symptoms. Two considerations render this supposition probable: the one, that in the class of diseases now more particularly under notice, the blood undergoes sensible alterations; and the other, that in constitutional contagious affections, properly so called, and which have so close an analogy with infectious disorders, it is difficult to imagine any other mode of origin than through a change in the circulating fluid."

"*Medical Constitution.*—The preceding remarks form a necessary introduction to a general notice of several remarkable modes in which infectious and contagious causes operate, or the circumstances under which they act upon a large number of persons. The first of these we will mention, is the medical epidemic constitution. This state has been erroneously defined the *appreciable* aggregate of meteorological conditions, during which diseases prevail epidemically. The phrase is synonymous with prevailing medical diathesis; it relates entirely to the peculiarities of the prevalent diseases, and incidentally alone, and that by way of explanation, to the existing state of the weather. It has been more correctly described as a general tendency, by virtue of which, nearly all diseases, at a particular time and place, no matter how different in seat and character, assume, to some extent, a common aspect. Thus, inflammations of the brain or bowels may both be complicated with bronchitis, which complication is then said to be the effect of the medical constitution. In like manner, when various diseases are attended with symptoms which indicate disorder of the liver, the medical constitution is said to be bilious, and we have bilious pneumonia, bilious pleurisy, bilious remittent fever, &c. So, too, the prevalent type of disease may be either inflammatory, adynamic, or ataxic; and thus it happens that an affection which, at one period, has been found amenable to a particular treatment, will, at another time, require an opposite management. Inflammations which last year may not only have borne, but seemed to require for their cure, the most profuse loss of blood, may, in the present, or the following year, be aggravated by moderate depletion, or even receive benefit from stimulating remedies. In connexion with this point, it may also be stated, that the alternate triumph and failure of the same remedy or class of remedies, has been observed even without there being any common feature in the diseases for which they were prescribed, or any, it would be more correct to say, which observation could detect.

"The medical constitution must not be confounded with epidemic disease, for the former is evinced by a common symptom or symptoms in numerous affections otherwise dissimilar to one another, the latter is a definite and peculiar malady. Yet during the prevalence of an epidemic, some one of its principal features is very apt to distinguish nearly all of the sporadic disorders which arise at the same time; so diarrhœa was an ordinary complication of most diseases during the prevalence of the cholera. In such cases it is fair to infer that the infectious cause of the medical constitution acquires unusual energy, not only capable of giving an impress to diseases generally, but of originating an independent malady."

This is all our author says on this subject.

Although, for the northern and eastern portions of our country, the omission to notice the malarial influence, as producing a peculiar medical constitution, may not be a matter of importance, yet, to the physician in the west or south, who sees, as a matter of daily observation, the effects of this wide-spread influence giving a caste to almost all the forms of disease that he encounters, it will seem very much like leaving out the essential point—like describing an ague and neglecting to mention its intermittent character. Indeed, to him, there is no point in pathology of more importance than this influence. In vain have we searched the work before us in hopes of finding, somewhere, a full account of this influence in modifying diseases of other types than intermittent and remittent fevers.

Excellent as we regard the work before us in most respects, we could not find a more striking illustration of the importance to students, who design practicing medicine in the west and south, of *there* studying the pathology of disease, than the omission to which we refer in this, the latest and best elementary work on pathology. We know that in mentioning this subject, we are treading on ground that has been ridiculed if not forbidden by the savans of the east; and assuming a position that is said to have been often refuted, &c., but we are told by our author, in his introductory essay, that "Three precepts may therefore be laid down as fundamental, and essential in all researches after medical truth: 1st. That the cases must be observed and recorded honestly, minutely, and fully. 2d. That they must be very numerous, the more so the better. 3d. That they must be comparable. It is very true that these conditions are not easily fulfilled; the labor, the humiliation of intellectual pride, the length of time, the weariness of a pursuit so nearly mechanical, are all obstacles in the way, and not easily surmounted. But there is no royal road to truth!" And if all this be true, and that it is, no one can doubt, then, when we have no pathological works setting forth, fully and accurately, the peculiarities of disease in an extensive region of country, the only opportunities for becoming acquainted with them are, to receive instruction from those who are familiar with those peculiarities, and by observing them personally. No one ought to contend that the general characters of a special disease are not the same; but all ought to admit, as our author abundantly teaches, that it may be modified, by various influences, so as to give it very different pathological aspects, and cause it to require very different modes of treatment.

What physician in the west does not know that pneumonia, here, is very apt to assume a decidedly remittent type, and, also, that, unless he adopts a different mode of treating such a case, from that he will find laid down by our eastern book makers for the treatment of pneumonia—a mode of treatment that shall, at the same time, combat the inflammation and jugulate the remittent fever—he will certainly have the privilege, if in Indiana, of laying in, as a preferred claim, his bill against the patient, "for attendance in his last illness." The same modifications, or similar ones, are found in connection with many other forms of disease—and modify their symptoms and the indications of cure.

But to return to our author. That he fully understands and admits the principle for which we are contending, is manifest from his remarks in reference to epidemic and endemic diseases. All we are sorry for is, that he has not recognized and treated, as ably as other points of pathology, the malarial influence and the diseased conditions arising from it.

"There is a feature, common to many epidemics, which must not pass without notice; it is the faculty which they have of taking the place, either in whole or in part, of the sporadic diseases which ordinarily occur in the regions where they prevail. It seems as if all morbid predispositions were, on such occasions, forced to assume the same mode of active development by the superior power of the epidemic influence. In a previous section the fact was mentioned, that if a number of persons were exposed to cold and humidity, many among them would be affected differently, according to the organ most predisposed to disease in each; conversely, in the case before us, it would appear that in the infected district nearly every one must have his constitution modified in the same way by the morbid epidemic principle, since the same form of disease results, whatever be the character of the exciting cause which directly produces it. It is on this account that, in estimating the mortality of a place during an epidemic, the number of deaths arising from ordinary causes, during a corresponding season in other years, are not to be added to those produced by the epidemic; for it not unfrequently happens that although it may have destroyed a large number of individuals, yet the total mortality from all causes may very little exceed the average of other years, so completely has the prevalent disorder become a substitute for the rest. We are accustomed to lament the insecurity of life in certain cities which are annually or periodically visited by epidemics, and to suppose their average mortality greatly to exceed that of places which enjoy an immunity from this evil; but it results from the vital statistics of these cities that their total mortality is not greater than that of others, and that if they suffer more from epidemic disorders, fewer of their inhabitants die of sporadic disease."

In part second, we have considered the type, duration, stages, terminations, and seat of disease.

Here, again, we find the continued, the intermittent, and remittent types referred to without an allusion to the very common case, in the west, of the continued disease being rendered both remittent and intermittent, as respects many of the essential features of the case, by the malarial influence.

In speaking of the terminations of disease, our author contends for the doctrine of critical phenomena, such as hemorrhages, sweating, certain states of the urine, effusions from the bowels, and other mucous surfaces, &c., as marking the terminations. Though his remarks are generally judicious, we think he attaches too much importance to these phenomena as signs. He even thinks the doctrine of critical days worthy of two or three pages.

The next point that comes up is, the theory of disease, and after a statement of the doctrines of the materialists, the vitalists, the humoralists, and the solidists, he closes the chapter with the following very judicious remarks, which we commend to the advocates of the different exclusive doctrines set forth:

"In the midst of these various theories of the origin of disease, the rational inquirer must be seriously embarrassed, if called upon to adopt some one of them exclusively; but if he has taken as his guide the rule of incorporating nothing into his scientific creed, but what results from a strict induction of facts, his perplexity need not be prolonged. He will then find that each theory rests upon a certain number of truths, but none upon a sufficient number to give it stability. He must speedily recognise the fact that a large number of the phenomena of life are explicable by chemical laws, and by them only, but that, at the same time, these laws are maintained in operation by the superintendence of vitality, a power which thus not only sustains them, but also upon certain occasion subverts them. He must also perceive that some functions are performed according to purely mechanical laws, but that their moving power is the principle of life, a force possessed of attributes different from those of gravity, elasticity, and the other mechanical powers. He must admit that, in some diseases, the starting point is in the solids, as where they are injured by violence; but he must, at the same time, acknowledge that not a few maladies called general, are directly owing to the introduction of some foreign matter into the blood-vessels, and do therefore originate in the fluids. He cannot deny that, even in the first-mentioned case, the blood speedily undergoes an alteration which is proportioned to the local hurt, and must, with better reason, be accepted as the cause of the general symptoms, than the local lesion itself; and that in the second case the original alteration of the fluids is usually followed by a more or less general change of structure in the solids. Finally, he cannot refuse to believe, that some affections commence by an impression upon the mind, the senses, or the general sensibility; but that amongst all of these, scarcely one remains confined to the system where it originated, but sooner or later implicates the rest. In a word, he beholds in man, a machine indeed, but one composed of various materials, which unlike those of other machines, are perpetually acting and reacting on one another; not only an aggregate of reacting elements, but one endowed with life, a power to which the ordinary laws of those elements are wholly subordinated, and whose own mode of being and laws are wrapped in profound mystery; not only a living animal, but a rational one, possessed of a soul, which, with peculiar faculties and susceptibilities, is capable of modifying the usual succession of phenomena resulting from the co-operation or conflict of the chemical, mechanical, and vital laws which govern the merely animal portion of the creature."

Chapter third speaks of general nosology. After a brief reference to the nosology of Cullen and Pinel, and the advantages of classification in affording facilities for studying disease, the author comes to the following conclusion, the correctness of which is generally admitted, and has caused the general neglect, of late, of nosological arrrangements in the study of disease.

"Yet, it should not be forgotten, that every nosological system is, to a great extent, artificial; and that it is rather an instrument for discovering truth, than in itself an expression of truth. None that has ever yet been devised, and it may safely be asserted, none yet to be invented, can so arrange diseases, that all shall be included in perfectly distinct groups. Some will be found which have an equally close relationship with several classes, and cannot, therefore, be included in a particular one, without a violation of the first principles upon which the classification is formed."

Chapter fourth treats of diagnosis, in which a mode of investigating and noting a case of disease is laid down which, if followed, would save much error; and its sad consequences, both on the patient and, in turn, the profession, would be avoided. The chapter goes on to give the modes of, and the means used in, physical examination. The following will show the manner in which the subject is treated:

"Pressure, which determines the *resistance* of a part by means of the hand, and palpation, or the act of handling, whereby is received whatever information the sense of *touch* can communicate, may be considered together, because usually associated in practice, although the one may be employed without the other. *Pressure* is applied with the whole hand, or with one or more fingers only, according to the extent of the surface examined, and the rapidity of the intended movement. It is employed to ascertain the degree of hardness in inflammation and œdema of the cellular tissue; the resistance of the walls of the abdomen, when distended by tumors, liquids, or gas; their relaxation after the evacuation of the contents of this cavity; and the presence of a solid body within a liquid abdominal effusion. An example of the sort last mentioned is presented by enlargement of the liver in ascites. By no other means can the projection of this organ below the false ribs be so well determined. The hand, or the ends of the fingers are pressed firmly over the point examined, and then thrust inward with a short, quick movement; if the liver lies near the surface, the layer of liquid between it and the integuments being suddenly displaced, the fingers impinge upon the solid viscus beneath. A similar movement communicated to the patella, in effusions within the knee-joint, will cause it to strike the condyles with a smart shock, and thus indicate the degree of the effusion.

"Pressure renders valuable assistance in determining the seat and degree of pain, and often detects its existence where the patient does not anticipate that it will be found. Some sorts of pain, those which depend upon inflammation, are aggravated by pressure in whatever manner it may be made, but others of the neuralgic and spasmodic kind are increased by pressure made over a small space, and usually soothed by the steady weight of the whole hand. The pain of abdominal inflammation, and that of colic, are with much certainty distinguished in this manner. Pressure, in like manner, may show a loss of sensibility on the part to which it is applied; pinching the integuments is the best mode of employing it for this purpose. It is also a valuable means of ascertaining the activity of the circulation in the extreme vessels; for when the blood moves languidly the pinkish colour of the skin returns but slowly after pressure made by the end of a finger is removed, while in scarlet fever, erysipelas, and other active cutaneous inflammations, the eye can scarcely follow the movement of the blood, so instantaneously does it efface the white spot made by the finger. When blood is effused by points in the skin, it is sometimes difficult to distinguish them from certain popular eruptions; but the latter always disappear on pressure, while the former become more distinct, from their contrast with the surrounding surface, which is rendered pale by its momentary loss of blood."

Palpation, or feeling for disease, the touch, the use of the speculum, succussion, percussion, auscultation, and the use of the microscope and chemical tests are brought severally under consideration.

We are inclined to think our author underrates the value of the microscope in detecting disease. The revelations which this instrument is making in reference to the physiological conditions and nature of the different parts of the body, seem to us clearly to point to great and important discoveries that are to follow from its use in the investigation of disease. The difficulties and uncertainties referred to may all have their origin in bad instruments, and awkward or careless observations.

The following are his remarks:

"The use of the *microscope* in pathological investigations is an art so difficult, that none but those who have especially, and for a long time, cultivated it, can depend upon the correctness of their observations, or estimate justly the phenomena they witness. On this account it can never become, like the methods heretofore discussed, of habitual employment in ordinary practice, even were the points of pathology which it is capable of elucidating much more numerous than they really are. The sphere of usefulness of the microscope is almost entirely limited to distinguishing the proximate elements of the body from one another, where these latter actually possess marked characters; but, according even to some of those most expert in its management, it is incapable of showing any differences between the particles of some very dissimilar bodies, such as pus and mucus, or tubercle and cancer. Without granting that such difficulties exist in the degree alleged, or that they are insurmountable, it must still be admitted that the value of the microscope is less questionable as a means of detecting crystallizable products in the urine, when they exist there in such small quantities as to elude chemical analysis; or animalcules in the sperm; or, when employed in the simple form of an ordinary magnifying glass, as a mode of detecting or identifying, the various minute insects and entozoa which infest the human body. Whatever pathological results have been obtained through the microscope will be mentioned hereafter."

The remarks of our author on the subject of which it has been satirically said

"Prognosis is the doctor's guessing,"

are appropriate, showing clearly the importance of it to the comfort and welfare of the patient and his friends, the difficulties attending it, and that it can only be elevated above the description in the Pindaric line above quoted, by a thorough acquaintance with diagnosis, and the nature and course of disease.

Preliminary to part third, on semiology, the symptoms and signs of disease are defined, the varieties of symptoms, and the value of semiolgy discussed.

Symptoms are the phenomena of disease simply, which may become signs when used to denote its existence or character. All facts that do this are signs.

"Symptoms are of various kinds. An important division is into *local* and *general;* the former manifesting themselves in some particular locality, the latter in nearly every part of the economy at once. To these may be added *sympathetic* symptoms, which are indeed local, but developed at some point distant from that primitively affected, and dependent upon its disorder for their existence. Thus in inflammation of the testicles, pain, redness, and swelling, are local symptoms, fever in general, and tumefaction of the parotid gland when it occurs, a sympathetic symptom. But, in strictness, there is no real difference between general and sympathetic symptoms, except that the former take place in systems which prevade the whole body, as the skin, the blood-vessels, &c., while the latter are evinced by organs of more limited extent. The connecting link between a local malady and its general symptoms is quite as obscure, as that which gives rise to the sympathetic disorders peculiar to any disease.

"*Premonitory* symptoms are those which usher in a disease, and which indeed form part of it; but, as several amongst them, such as chill, fever, debility, headache, &c., are common to the invasion of numerous maladies, they are usually spoken of as if they did not belong to the attack which is about to follow.

"A *Pathognomonic* symptom is one that is characteristic of a disease, and, although alone, indicates its nature, because it exists in no other affection. Symptoms of this sort are very few indeed, and in every case, perhaps, consist in some *physical* accident or attendant of the malady. The discharge of worms is of course a certain sign of verminous disorder; that of false membrane from the larynx, of croup; that of calculi from the bladder, of lithiasis; the detection of the acarus scabiei is conclusive of the itch; &c. But there are, in each of several affections, a few symptoms which taken together may be regarded as characteristic, or so strongly indicative of a particular disorder as to leave but little room for doubt. An acquaintance with these is all-important in practice, since it at once leads the physician to a probable diagnosis, and directs him to the particular points upon which his subsequent examination should chiefly bear. Thus if he find that there is cough, stitch in the side, rusty and tenacious sputa, and crepitant rhonchus, he can have but little doubt that the case is one of pneumonia; or if he observes fear, præcordial oppression, and a rubbing or to-and-fro sound over the heart, he may feel assured that he has before him a case of pericarditis. Just in proportion to the minuteness with which smyptoms are studied, and the accuracy of the value assigned to them, will these characteristics of disease become more and more certain, and diagnosis be divested of its difficulties."

Chapter first treats of signs from the exterior of the body as manifested by the position, color, temperature, &c.

In reference to cold, our author says:

"A chill occurring in the course of a disease which is not paroxysmal, is generally a sign, either of an intercurrent inflammation, or of the occurrence of suppuration in a part already inflamed, and is, therefore, of evil augury. Thus, in the progress of continued fevers, such an occurrence renders it probable that an attack of pneumonia, pleurisy, pericarditis, endo-carditis, or meningeal inflammation, is about to take place; during the decline of typhoid fever, a severe chill is usually accompanied with abdominal pain, and is then a sign of peritonitis from perforation; in the puerperal state, this phenomenon may accompany the establishment of the lacteal secretion, or, if the abdomen become tender at the same time, metro-peritonitis may be anticipated; at a somewhat later epoch, it is more likely to threaten an attack of phlegmasia dolens, or the formation of an abscess in the mamma. After wounds, a rigor may precede the appearance of erysipelas, or inflammation of the veins, or the ordinary consequence of traumatic phlebitis, the formation of purulent deposits in the lungs, brain, liver, or other viscus. In the course of all parenchymatous inflamations, a chill denotes the advent of the suppurative stage. This is seen in simple phlegmon, and in pneumonia. A like occurrence in phlegmasiæ of the serous membranes is said to announce that pus instead of serum has begun to be secreted. The daily recurrence of a slight chill, followed by fever and sweat, when it takes place in a chronic affection marked by debility and wasting, is called *hectic* fever, and if it attends organic disease, shows that the fatal issue of the attack is approaching. It does not, however, follow that hectic is always a fatal omen. It may occur in chronic bronchitis, in empyema, and in large external suppurations, and yet the patient recover."

Here, again, we find the important omission to notice that, during the course of various diseases in malarious districts —and that not occasionally but frequently—"a slight chill followed by fever," daily, or on every other day, may occur as the effect of the malarious influence, which, according to the above, would be regarded as evidence of suppuration or hectic fever.

We have now under treatment a patient who was, a few days ago, attacked with a violent inflammation of the right lung, which was subdued by free bleeding, nauseating, cathartics, diaphoretics, anodynes, blistering, &c., but, as the fever and pain subsided, and the patient was able to take a full inspiration with less suffering, there occurred each day, at about noon, a sensation of chileness, followed by an increase of the febrile action, that lasted until the latter part of the night.

We gave her a few doses of quinine in addition to the treatment indicated by the pneumonia, and she is rapidly convalescing.

We do not mention this as a rare occurrence, for most of our readers witness such cases continually; but simply as an illustration.

We next have the signs from the head, face and neck. We quote the entire clause, in reference to the eyes.

"*The eyes and their appendages.*—The eyelids, like other muscular parts, are feebly moved in low fevers, and nearly conceal the ball of the eye: they not only hide it, but strongly oppose being opened in those forms of ophthalmia which are attended with intolerance of light. In paralysis of the portio dura, the orbicularis ceases to contract, the lids remain open, and expose the parts beneath to the irritation of the air and dust, which soon excites severe inflammation. Paralysis of the third pair deprives the upper lid of motion, and effectually shuts out light from the eye. If this condition co-exist with loss of power on the opposite side of the body, cerebral disease may be inferred. Otherwise it may be owing to a blow upon the superciliary ridge, or to rheumatism of the levator muscle, or to a tumour or other disease within the orbit. An open state of the eyelids in sleep may be owing to spasm of the levatores; some writers have attributed it in children to verminous disease, but many individuals in perfect health habitually sleep with their eyes half open.

"The tears, instead of being absorbed by the punctæ lachrymales, may run down upon the cheek. This indicates, usually, obstruction of the lachymal duct. It occurs at intervals, when the nerves around the eye are attacked with neuralgia; commencing inflammation of the conjunctiva, also excites a profuse flow of tears. The accumulation and hardening of the palpebral mucus upon the edges of the lids indicates an extreme degree of prostration.

"The expression of the eye, and its changes in position, form, color, brightness, and power of vision, possess a very decided interest in connection with the present subject. The eyes are unusually bright during high fever with maniacal delirium, but dull and hazy in low fevers, and all affections in which there is sluggishness of the intellect or stupor. The cornea becomes still more completely clouded upon the approach of death, and often flakes of mucus partially obscure its surface. Immobility of the eyes may depend either upon paralysis of their muscles, or upon nervous spasm. It rarely happens that both eyes are paralyzed, except from extensive disease within the brain; cerebral disorganization, whether occurring near the orbit, or in the neighborhood of the roots of the third, fourth, and sixth nerves, is more apt to paralyze the muscles of only one eye. Fixedness of the eyes is observed in some inflammatory affections of the brain, along with rigid and open lids, and constitutes what is called *ex-trasis;* in cataleptic fits the eyelids remain open, and the eyes, like the rest of the body, are immovable. In epilepsy, chorea, and other true convulsive diseases, the movements of the eyes are rapid, irregular, and often distorted.

"Squinting, or *strabismus*, besides being natural, or acquired by imitation, and so far of no importance as a sign, may depend upon disease within the cranium, or in the orbit. Conjoined with cerebral symptoms, it is of most unfavorable significance in adults, but far less so in young children, in whom squinting may arise from various causes unconnected with disease involving life. Tumors, &c., of the orbit often produced strabismus either by pushing the ball of the eye to one side, or by paralyzing a portion of the muscles. Rheumatism of those parts occasionally induces squinting. In a case of the sort under the writer's care, the eye did not regain its natural position for several months.

"Morbid prominence of the eye depends upon the turgid state of structures lying behind it, and often conveys a deceptive impression of the size of the organ. Protrusion of the eye, when gradual and permanent, is a sign of a tumor developed behind the eyeball, as enlargement of the lachrymal gland; encysted, fatty, bony, or cancerous tumors; aneurisms, and other tumours extending from the cerebral, nasal, or maxillary cavities, into the orbit; an effusion of blood behind the ball; and finally, inflammation of the cellular tissue at the bottom of the orbit. The eyes are hollow and sunken in all cases of general emaciation, owing to the removal of the layer of fat at the bottom of the orbit, and is of no further significance than as it marks the progress of emaciation. It is frequently very striking after sudden and exhausting discharges, such as diarrhœa and venereal excesses, although the general bulk of the body may not seem to be diminished.

"Vascularity of the conjunctiva frequently accompanies inflammation and congestion of the brain, but is more strongly marked when the membrane is itself inflamed. The sclerotic coat is usually the part which first becomes yellow in jaundice; in phthisis it acquires a pearly whiteness. In rheumatic ophthalmia and irritis it is marked by a radiated redness around its junction with the cornea. Ulcers of this latter tissue are most apt to occur in scrofulous persons, either from direct injury or after small-pox, and to leave behind them opaque cicatrices. Discoloration of the iris, and a fibrinous deposit upon it, with irregularity of the pupil, are signs of iritis. In the syphilitic form of this inflammation the pupil is said to be drawn upwards and inwards.

"The humours of the eye undergo a real or apparent change of color in several diseases. The equeous humor may be mingled with pus; the crystallie become opaque and of a white or buff color; and the vitreous humor sometimes appears green. The last-named hue is merely apparent, and is believed to be caused by the reflection of the blue of the choroid coat through an amber-colored lens, these two colors by their union producing green. The pupil is generally dilated when sight is impaired by imperfection of the visual power, or by central opacity of the lens. The former state attends the various forms of amaurosis, and many cases of diseased brain without active inflammation. Exaltation of the functions of the brain and of the retina, most commonly occasions contraction of the pupil. The action of certain medicines on the pupil should be kept in mind. It contracts under the influence of opium, but is widely dilated by stramonium and balladonna."

The signs from the digestive apparatus are admirably discussed; and as it is the universal custom of physicians in examining patients to look at the tongue, principally to see whether it is much coated or not, and as we believe but little evidence is given of the existence or character of disease from the coating, we quote what our author says in reference to it:

"The tongue is *pale* whenever the blood has lost its normal proportion of red globules, as in hemorrhage chlorosis, in chronic inflammation also of the intestinal canal, unattended with much fever. A bright *red* color of the tongue is found in all violent inflammations, but especially in those of the upper portion of the digestive tube. Sometimes, as in scarlatina, the redness is not uniform, but shows itself in disseminated points, which are the turgid papillæ, projecting through a white coating of mucus; or this color may be confined to the sides and tip of the organ, while its centre is whitish. A moderate coating, whether white or yellowish, has generally been supposed to indlcate accumulation of mucous or bilous matters in the stomach, and a red, dry, and glazed tongue, chronic inflammation of the intestinal canal. But these signs, as well as most of those derived from the presence, or the peculiar characters of the coating, are very fallacious. M. Louis found that redness of the tongue existed with equal frequency in phthisical patients who had no lesion of the stomach, and in those of them in whom this organ was extensively diseased. A similar result was arrived at by this author in regard to the state of the tongue in typhoid fever.

"The gradual disappearance of the coating from the circumference towards the centre of the tongue, is a favorable sign, and, in acute diseases, marks the commencement of convalescence. This process is sometimes arrested, and the tongue becomes dry and red upon the occurrence of some inflammatory complication, but the change may also depend upon too great strictness of the patient's diet, for the more favorable condition has often been found to return upon the cautious administration of more nutritious food. The tongue, like other parts within the mouth, may become the seat of membranous formations; whenever this occurs as a complication, either in acute or chronic disorders, and along with great exhaustion, it is a most unfavorable sign. Ulcers of the various sorts above described, aphthæ, and the postules of small-pox, are also met with upon the tongue."

We next have a very judicious account of the signs of disease furnished by swallowing, the appetite, nausea, vomiting, defacation, &c. In reference to the last mentioned, the following will probably bring to mind a frequent neglect on the part of almost every practitioner:

"The inspection of the *alvine evacuations* is of great utility in practical medicine. It is too much the habit, at present, to omit their examination altogether, on account of its repulsiveness, and the difficulty of effecting it, either in private or public practice; and this habit has induced the still more blameable one, of neglecting to make proper inquiries on the subject."

The signs derived from the examination of the alvine evacuations are given under the heads of consistence, color, serous or mucous character, evidence of digestion and lientery, odor, &c.

The genito-urinary apparatus gives some important signs of disease; but of these, those derived from the urine are, by far, the most important in reference to which our author is full and explicit. We can give but the following brief summary:

"To complete this portion of the present subject, it remains to point out the semeiological value of the symptoms which have been described. Profuse and chronic discharges of urine, containing sugar, are characteristic of diabetes; a like abundance, of a nonsaccharine fluid, indicates the disease variously denominated diabetes, insipidus, hydruria, and polydipsia. Clear and transparent urine, containing neither blood nor mucus—frothing when agitated, and coagulating on the application of nitric acid and heat—is albuminous, and, in chronic disease, indicates granular degeneration of the kidneys. During the first stage of Bright's disease, the urine contains blood corpuscles. Urine made green by the addition of nitric acid, contains the coloring matter of the bile. Crystals, a gritty sediment, and calculi, in the urine, announce the existence of some one of the forms of gravel, which is to be determined more precisely by the methods above described. Hydatids, in this fluid, generally proceed from a cyst in the kidneys.

"Mucus in the urine, if in considerable quantity, is generally owing to visical catarrh, or to enlargement of the prostate gland. Pus may be derived from inflammation of the pelvis of the kidney, (*pyelitis,*) or from that of the lining membrane of any part of the urinary passages, or from an abscess of the kidney, or postate gland; and when sanious, fœtid, and bloody, it is almost certainly indicative of malignant disease of the bladder. Blood in the urine is commonly due either to inflammation of the kidney, to calculous concretions in its pelvis, or to erosion of a vessel in the bladder. It also frequently accompanies the course of a variety of acute and dangerous diseases. The urine in small-pox, for instance, is often bloody. So is it in purpuro, in typhoid fever of bad type, in scorbutus, in acute anasarca from exposure to cold, &c. It may even occur as a distinct affection, without any general or associated symptoms. The manner in which pus and blood are voided, is of consequence in diagnosis. If they are discharged with the commencement of the stream, it may be presumed that they are formed in the urethra. If they accompany the conclusion only of the discharge, they are usually regarded as proceeding from vesical disease, and, if intimately mixed with the urine, from the kidney. But it is evident that the value of this distinction is doubtful; for, whether the bloody or purulent discharge proceed from the bladder or the kidney, it will, if the patient remain at rest, be voided only at the conclusion of urination; and if he be in motion, it will mix with, and accompany in its emission, all the urine contained in the bladder. Bodies, looking like earth-worms, are sometimes passed from the bladder. They are coagula of blood, moulded in the ureters. They generally give rise, while in these canals, to the symptoms of nephritic colic, or of a fit of the gravel.

Frequent calls to urinate may be owing to a large increase of the renal secretion, and, when attended with pain, to a concentrated state of the urine,—to inflammation of the bladder—the ingestion or absorption of the active principle of cantharides, or of turpentine; to the presence of a calculus in the bladder, to enlargement of the prostate gland, to pressure of some adjacent organ or tumour, and to an excited state of the nervous system. Infrequency of voiding the urine depends, in disease at least, either upon its scanty secretion, (ischuria, suppression) or its retention in the bladder. Two forms of the latter are to be distinguished: the one in which a physical obstacle prevents the escape of the urine, and the other in which the patient's sensibility is so blunted that he is unconscious of the distention of his bladder. In the latter case, when a certain amount of urine is collected, all beyond that quantity forces a passage, and there seems to be incontinence, when there is in reality retention. In paraplegia, the amount thus retained becomes gradually less and less, and at last the urine escapes from the urethra almost as fast as it is poured into the bladder. Such incontinence is also not unusual after operations for the removal of stone in the bladder, especially when the patients are very young. A twisted, forked, or thin stream of urine, is commonly indicative of stricture of the urethra; it may, however, be caused by a fragment of stone, or other foreign body, in this canal."

We have already devoted so much space to the work that it will be impossible to notice the remaining portion as fully as that we have given.

The signs derivable from the nervous system, are both interesting and important; but many of them are quite vague and uncertain.

Those derived from the circulatory apparatus are, after a brief reference to the physiology of the heart, arteries, and veins, considered in relation to their impulses and auscultatory sounds.

The last apparatus, and it is one from which the signs are well laid down, is the respiratory.

Part fourth, on general morbid anatomy, is introduced by rules for making and noting post mortem examinations.

This is a most important matter; for so very few of our physicians are acquainted with it that, when *called upon* to make such examinations—for they scarcely ever ask the privilege—they know so little of the proper mode of conducting it, that it would about as well be omitted, for any information that they gain by it.

The rules are too lengthy for an insertion here, but ought to be observed by all physicians who make examinations with a view of deriving information, in morbid anatomy, from them.

We extract, from morbid anatomy of the blood, the following:

"Pregnancy, although a physiological condition, induces remarkable changes in the composition of the blood. It has long been known, that a degree of plethora sometimes accompanies this state, which requires, for it is relieved by, depletion. When, however, the blood of pregnant females came to be examined, it was found to be generally impoverished: that is, to contain a diminished proportion of globules. The same observers who determined this fact, also concluded from their experiments in cases of plethora unconnected with pregnancy, that in plethora the proportion of globules is increased, and that the symptoms of the affection depend upon this increase, and not on any augmentation of the mass of the blood, as was previously believed. They found, therefore, the fact, that pregnant females are subject to symptoms of plethora, one of difficult solution. As will be presently shown, the proportion of globules in plethora is *not* increased, and the plethoric symptoms depend upon over-distention of the vessels. This distention may take place under any circumstances of the composition of the blood; and that it does occur in pregnancy, is abundantly proved by the turgidness of the vessels and all the tissues, the headache, disorders of vision, heaviness, vertigo, &c., which sometimes attend it. But when blood is drawn from a pregnant female, it is found that the mean proportion of the globules does not exceed 112-6, and that this change becomes more marked as gestation advances. At the same time, the quantity of fibrin either remains normal, or is slightly augmented. Pregnancy, then, is allied to plethora as well as to anemia. In it these two conditions may actually coexist, if the meaning of the latter is restricted, as it usually is, to a deficiency in the proportion of globules in the blood.

"No matter what the disease in which the blood has been examined, this fluid has uniformly been found to undergo some change. The proportion of fibrin varies according to the nature of the disease; but, independently of this condition, certain changes rarely fail to take place. These are, a marked diminution of the globules; one, less in degree, of the albumen of the serum; and an increase of the fatty matters, particularly of the cholesterin. It is not easy to decide whether these alterations of the circulating fluid are owing directly to the disease in every case, or to the diet which, in acute affections, cuts off the supply of nutritive materials, and, by suspending digestion, makes smaller demands upon the liver, and allows the cholesterin to be retained in the blood. In chronic maladies, alterations of the blood take place more slowly, but are, on the other hand, more complete. In either class of disorders, the changes are greatly hastened by depletion; after each bleeding, the blood grows more watery, and its globules are sensibly diminished, but its fibrin undergoes no change. Becquerel and Rodier give the average quantities of the several elements of the blood in persons who had been bled three times. In these cases the proportion of globules fell from 129·2 to 99·2.

"The authors just mentioned have established, by their experiments, that the *plethoric condition*, and the symptoms incident to it, are probably the effect of an increase in the mass of the blood, and in nowise of any change in its composition. The average proportion of globules found by them in six cases of well-marked plethora, in males, was 138, or rather less than the normal average. MM. Andral and Gavarret found the average, in thirty-one plethoric persons, (sex not mentioned,) to be 141, or precisely the average for healthy blood obtained by Becquerel and Rodier. If, then, this latter number be admitted to be correct, it is clear that the blood undergoes no change of composition in plethora, and the symptoms of the disorder can only be referred to the excessive quantity contained in the vessels."

The following, in reference to the fluid of dropsies, is interesting, being, in part, new:

"The fluid of dropsical effusions is not the same in all cases. Sometimes it contains a large proportion of dissolved fibrin, which, coagulating, forms false membranes in cavities, and an interstitial deposit in parenchymatous organs; sometimes, on the other hand, the fluid is simply serous. The cases of the former kind are nearly all due to inflammation, and constitute the class of *fibrinous dropsies*. The effused fluid, as stated, generally coagulates within the body, but it sometimes preserves the liquid form until removed artificially, and then coagulates. This fluid is supposed to be extravasated chiefly by the capillary vessels, while that of *serous*

*dropsy* permeates the walls of the veins almost exclusively. These two forms have been only of late distinguished from one another.

The fluid of dropsy was formerly supposed to be identical with the serum of the blood, but the analyses of MM. Andral and Gavarret have shown the incorrectness of this opinion. In sixteen cases of serous dropsy examined by them, the highest proportion of albumen in the effused liquid was 48, and the lowest 4, the average proportion of serum in the healthy blood being 70. These observers did not find that the *seat* of the effusion made any sensible difference in the proportion of albumen. The state of the constitution, however, influenced it; the less the strength of the patient, the less the albumen, and *vice versa.* Thus in six cases of hydrocele, the subjects of which were all in robust health, the proportion of albumen varied between 35 and 59; and when, in dropsy of the larger cavities, tapping was performed several times, it was found that the quantity of albumen in the fluid evacuated progressively declined. The proportion of water in dropsical effusions was found to vary between 950 and 986, the normal average in the blood being 790. All of the cases of dropsy here alluded to were due to venous congestion from mechanical causes. Had they arisen from inflammation it is probable that the proportion of coagulable matter in the effused fluid would have been greater; since, as the authors alluded to remark, it is found to be so in the fluid obtained from the skin by vesication.

"The preceding statements furnish the positive proof of what has long been alleged, that the blood is impoverished in dropsy, and show that its impoverishment is due to a loss of albumen, and in some cases of fibrin. Confirmatory negative evidence of this proposition is to be found in the fact that a decline in the proportion of neither of the remaining chief constituents of the blood, fibrin or red globules, without that of albumen, ever produces a similar result, for dropsy does not exist either in chlorosis, or in fevers and affections of a typhoid type, except as an unusual complication."

Passing over the pathological anatomy of inflammation, lesions of nutrition, and morbid growths, we come to the closing paragraph on the subject of parasites:

"*Parasites.*—Certain independent organisms, both vegetable and animal, are found in the human body. The vegetable growths are all microscopic, and belong to the lowest orders of plants, the *algæ* and *fungi.* They are never met with except upon cutaneous or mucous surfaces, nor while these surfaces remain healthy. Usually, a secretion of fibrin or mucus, undergoing decomposition, forms the soil in which they grow. In some cases, they are believed to be the media of contagion, as in that form of impetigo which affects the hair-bulbs of the scalp, and is called *favus*. This is the most important example of their connecion with disease.

"Animal parasites are very numerous. Many of them are infusorial; many belong to the class of insects and mites, as fleas, lice, bugs, and the acari, of which the most important one is the *itch-mite*. A class of higher consequence comprises several sorts of worms. Those which infest the intestinal canal are extremely common, and are the *oxyuris vermicularis*, or *thread-worm*, which inhabits the rectum; the *trichocephalus dispar*, or *long thread-worm*, which is found in the large intestine, and especially in the cœcum; *ascaris lumbricoides* or *round-worm*, whose ordinary residence is the small intestine; and the *tape-worm*, or *tænia*, which also affects the same part. The kidney is occasionally the seat of a round-worm, called the *strongylus gigas*, measuring from five inches to three feet in length, and from two to six lines in thickness. For a detailed account of these animals, of the *trichina*, the *echinococcus*, of *acephalocysts*, and numerous other parasites of still inferior interest, pathologically, the reader must consult special treatises.

"The diseases with which even large intestinal worms are connected, appear to be sometimes the cause and sometimes the effect of the presence of these parasites. Very often they exist in considerable numbers without producing the least disturbance of the economy, but in other cases they are unquestionably the cause of much suffering and ill health. How far they are themselves the result of a morbid state of the organs in which they appear, is still an undecided question.

"The origin of parasites is extremely obscure, and has long been a mooted point among naturalists. It may not be inappropriate to present a summary of the opinions which are entertained respecting a subject of so much interest, but in doing so, we shall confine our remarks to the parasitic animals which inhabit the interior of the body, or *entozoa*.

"It is evident that these animals must originate in one of two ways; that they must be derived directly or indirectly from without, or be created out of the materials existing within, and furnished by, the body. No other supposition is possible. If an entozoon is in any manner derived from without, it must be admitted that this takes place either through the reception of the animal itself, or of its ova. If either opinion be assumed, it follows that the parent animal must exist somewhere external to the body. But the parasites in question have never, in any case whatever, been detected except within the organism. If it is objected that many of these animals are so minute that they might easily elude discovery in the elements around us, the argument fails when applied to the giant strongylus, the stout lumbricoid worm, and the tænia measuring many yards in length. Besides, even admitting for a moment the possibility of the parasites which inhabit the intestine, and other mucus cavities, having once existed externally, the insuperable difficulty still remains of explaining the entrance of entozoa into shut cavities and parenchymatous structures, into the eye, or the muscles, for example, and their presence in the unborn child, and even in the bodies of larger entozoa of a different species. On the other hand, if it is maintained that the ova are alone received, it must still be shown that the ova exist external to the body, which has never been done. Nor would the admission of this explanation be sufficient; for many of the entozoa are not propagated by eggs, but belong to the viviparous class, so that in regard to them the difficulty remains undiminished. But granting the existence of ova without, and their reception into the body, it is still impossible to explain the development from them of the animals found in the parenchymy, in the embryo, &c., without at the same time admitting that the ova are not only carried to these localities through the blood-vessels, but actually pass through the walls of the capillaries. Such an admission would be a physiological absurdity; for the extreme vessels will allow of the passage of a single blood-globule, at a time and no more, and will not permit any denser fluid than the plasma of the blood to permeate their walls, how then could they afford a passage in any manner to ova, the least of which is ten times as large as a blood-globule?

"If the hypothesis now presented is untenable, it only remains to adopt the alternative one, to-wit, that entozoa are generated or created anew out of the materials or the products of the living organism. It may be urged affirmatively, in support of this doctrine, that each organ possesses its own entozoa; the kidney, a species different from those of the intestine, which are, again, unlike the parasites of the liver. Even more, the several parts of the same organ generate dissimilar animals. The small intestine produces the round and the tape worms, the large intestine the two species of thread worms. These facts seem to show that some extremely local concurrence of circumstances is essential to the production of the several entozoa. It may also be argued, and we think the argument unanswerable, that if spermatic animalcules, which exist in the testicle, are there spontaneously generated, no violence is done to probability in supposing parasitic animals to be produced in the same manner It will hardly be denied that spermatozoa are literally evolved from the constituents of the semen. But it is objected to the doctrine of spontaneous generation that it is against analogy, which everywhere supports the famous dogma, *omne vivum ex ovo*. This objection is a mere begging of the question. The decision of the case in hand involves the truth of the theory just quoted, and as we believe, must be allowed to show that this theory is not absolutely universal in its application. Other facts, also, amongst which are the following, tend to invalidate it. Nothing can be more certain than that all organized beings were, at some time or another, created; geology proves that successive genera and species have been thus created, at long intervals apart; and the history of disease renders it probable that one affection at least, syphilis, which is now propagated only by direct descent, *ex ovo*, as it were, is really of comparatively recent origin.

In conclusion, after a review of the preceding outline of an argument upon the generation of parasitic entozoa, we feel obliged to admit that the weight of facts and probabilities is wholly on the side of the doctrine of spontaneous generation; at the same time, we cannot but look with interest to the results of future observation in this field, nor altogether suppress the hope that the simple law of nature, *omne vivum ex ovo*, may even yet be found to embrace the classes which now appear to form so striking an exception to its provisions."

We have thus given some idea of the work and its different parts, but hope our readers will not be satisfied without the work itself, and, in fact, the whole library of which it is a volume. The publishers send direct by mail, when ordered, the works of the series, as fast as published. E.

